# 

**Published:** 1904-12-31

**Authors:** 


### NOTICE TO CORRESPONDENTS.

All MSS., Letters, Books for Review, and other matters intended for the Editor should be addressed The Editor, "The Hospital," The Hospital Buildings, 28 & 29 Southampton Street, Strand, W.C.

The Editor cannot undertake to return rejected MS., even when accompanied by stamped directed envelope.

**Notice to Advertisers and Subscribers.**

All Advertisements, Orders for Copies of The Hospital, and Business Communications should be addressed to **THE MANAGER (Not to the Editor)**, The Hospital, 28 & 29 Southampton Street, Strand, London, W.C.

The Cost of Subscription, post free, is as follows:—Per week, 3½d.; per quarter, 3s. 3d.; per half-year, 6s. 6d.; per annum, 13s. Foreign and Colonial:—Per annum, 19s. 6d., payable, in all cases, in advance.

**NOTICE.—Vol. XXXVI. of "The Hospital" (April to September 1904), in handsome cloth binding, is now on sale at the office of this Journal, price 10s. 6d. Cases for binding the Numbers contained in this Volume 2s. Index to Vol. XXXVI., 3½d., post free.**

**The Monthly part for December is now ready. Price 1s., by post, 1s. 3d.**

### Medical Appointments.

George Adams, M.A., M.B., Ch.B.Aberd., House Physician, Royal Aberdeen Hospital for Sick Children. **T. Crisp English**, B.S.Lond., F.R.C.S.Eng., Lecturer on Operative Surgery, St. George's Hospital Medical School. **F. A. Field**, M.D.Lond., Honorary Anæsthetist to St. Paul's Hospital, Red Lion Square, W.C. **J. L. Forrest**, M.B., Medical Officer of Health, Marshland Rural District. **D. P. Gage**, L.R.C.P.Edin., L.F.P.S.Glasg., Certifying Factory Surgeon for the Kilwinning (Burgh) District, Ayrshire. **John Gutch**, M.D., B.C.Cantab, Member of the Honorary Medical Staff to the East Suffolk and Ipswich Hospital. **C. Holding**, L.R.C.P. and S.Edin., District Medical Officer Whitchurch Union. **A. P. Luff**, M.D., F.R.C.P.Lond., a Member of the In-patient Staff, St. Mary's Hospital. **Thomas D. Luke**, M.B., F.R.C.S.Edin., Lecturer on the Practice of Anæsthetics at the University of Edinburgh. **G. B. Picton**, M.B., B.S.Durh., District Medical Officer of the Redruth Union. **O. H. Rogerson**, L.S.A., District Medical Officer of the Northallerton Union. **J. B. Ryan**, L.R.C.P. and S.Irel., Certifying Factory Surgeon for the Taghmon District, Wexford. **J. F. Sheppard**, L.R.C.P. and S.Irel, Certifying Factory Surgeon for the Newport Pagnall District, Buckingham. **A. Smith**, M.D., Medical Officer of Health, Whickham Urban District. **F. Sugden**, M.B., District Medical Officer of the Doncaster Union. **H. L. Spark**, M.B., B.S.Edin., District Medical Officer of the Bradford Union.

**"The Hospital" Institutional Library.**

**"Hospitals and Asylums of the World." 4 Vols., with a Portfolio of Plans, complete £12 12s.**

**"Burdett's Hospitals and Charities, 1904." 5s. net.**

**"Burdett's Official Nursing Directory." 8s. net.**

**"Cottage Hospitals, General, Fever, and Convalescent." 10s. 6d.**

**"Uniform System of Accounts for Hospitals and Public Institutions." New Edition, 4s. net.**

**"Hospital Expenditure: The Commissariat." 2s. 6d.**

**"A Legal Handbook for the Use of Hospital Authorities." 2s. 6d.**

**"The Cottage Hospital Case Book or Register of Patients." 10s. and 12s. 6d.**

**All these are published by the Scientific Press, Ltd., and may be obtained through any bookseller, or direct from the publishers, 28 and 29 Southampton Street, Strand, London, W.C.**

### Medical Appointments.

### IMPORTANT NOTICE.

A Useful New Year Present for Nurses.

THE

### HOSPITAL

### Calendar & Notebook

FOR

### 1905.

A Useful New Year Present for Nurses.

**This compact and useful little Notebook, which is of a convenient size for the pocket, is in appearance an exact reproduction of THE HOSPITAL Journal in miniature and contains much information of value to nurses including: Hints for nurses; How to advertise; How to reply to an advertisement; Hints on how to accept a post; Measures; Apothecaries' and other weights, &c., &c.**

### To Nurses.

THE HOSPITAL Calendar and Notebook will be forwarded **Post Free** on receipt of **Two Penny Stamps.** Nurses requiring additional Copies can obtain One Dozen, post free, for Nine Penny Stamps.

**BINDING CASES,** **in New Pegamoid Leather Cloth, gilt lettered, specially designed to take THE HOSPITAL Calendar and Notebook, with loop for hanging from Wallet or Chatelaine with One Copy of the Calendar, 3½d. post free. These Cases are strongly made, and can be used from year to year, as they do not contain the year nor any lettering other than the title of the Calendar.**

The Diary will shortly be ready, and it is advisable to make immediate application to avoid disappointment, as the edition is limited and no reprints will be issued when it is exhausted.

*Address*—The Manager, Calendar Dept., THE HOSPITAL,
28 & 29 Southampton Street, Strand, London, W.C.

